# The updated surgical steps of gasless transaxillary endoscopic thyroidectomy with neck level and region orientation for thyroid cancer

**DOI:** 10.3389/fonc.2024.1377878

**Published:** 2024-05-10

**Authors:** Yuqiu Zhou, Chunyan Shui, Linjie Ma, Yongcong Cai, Ronghao Sun, Jian Jiang, Dingfen Zeng, Xu Wang, Xiaoli Xu, Pei Huang, Chao Li

**Affiliations:** ^1^ Head and Neck Surgery Department, Sichuan Clinical Research Center for Cancer, Sichuan Cancer Hospital & Institute, Sichuan Cancer Center, Affiliated Cancer Hospital of University of Electronic Science and Technology of China, Chengdu, China; ^2^ Operation Room, Sichuan Clinical Research Center for Cancer, Sichuan Cancer Hospital & Institute, Sichuan Cancer Center, Affiliated Cancer Hospital of University of Electronic Science and Technology of China, Chengdu, China

**Keywords:** thyroid cancer, surgical procedures, complications, surgical trauma, endoscopic thyroid surgery

## Abstract

**Introduction:**

We previously made a detailed expansion to the gasless transaxillary endoscopic thyroidectomy(GTET) procedure described in the previous literatures. In this study, we optimized the procedure focused on the limitation of the approach in terms of trauma and lymph node dissection and made a comparison with the early procedure.

**Materials and methods:**

This paper gave a detailed description of the updated procedure and prospectively collected data about patients with papillary thyroid carcinoma(PTC) performed by the two procedures from December 2020 to April 2023. The differences in surgical outcome, surgical trauma and parathyroid gland(PG) function protection were analyzed.

**Results:**

Of the 302 patients, 184 underwent with early procedure(EP), and 118 underwent with updated procedure(UP). The surgical outcomes of operative time, time of thyroidectomy and central neck dissection, blood loss, drainage and postoperative hospital stay were shorter in UP than that of the EP. The mean number of lymph nodes retrieved and weight of dissection lymphatic tissue in the UP were significantly more than that in EP without increasing the mean number of metastatic lymph nodes. Postoperative complications did not differ between the two procedures. The UP had more advantages in the identification and preservation of the superior parathyroid gland, however, it did not improve the preservation *in situ* of the inferior parathyroid gland. The visual analog scale score for pain and the changes among inflammation factors was lower in the UP.

**Conclusion:**

The UP of GTET could perform safely and efficiently while reducing surgical trauma in selected patients.

## Introduction

The transaxillary approach is one of the most popular remote-access thyroid surgery approaches first described by Ikeda in 2000 (1) and then it was modified by Chuang in 2006 by using a gasless system (2). At present, it is reported to be the most widely used remote-access thyroid surgery approach (3, 4) with the advance of clear vision, convenient operation, safety and cosmetic satisfaction. With the trend, we previously made a detailed expansion (5) to the gasless endoscopic surgical procedures based on literatures described previously (6–9) and summarized the steps for indication-based selected patients with thyroid cancer, then promoted the technique in our country. However, the approach of gasless transaxillary endoscopic thyroidectomy(GTET) has its limitations. It is not comparable to conventional open thyroidectomy in terms of lymph node dissection (10), and the invasiveness of the working space of GTET is the greatest compared with other remote access thyroid surgical approaches (11). Currently, the improvements to limits have not been described in the published articles. Meanwhile, our previous article mainly focused on familiarity with surgical procedures and how to perform them safely but lacked an in-depth discussion of the above aspects. Therefore, this article aimed to optimize the surgical procedures, especially in terms of trauma and lymph node dissection based on our previous steps and high volume of cases to strengthen the concept of radical treatment of thyroid cancer and improve the efficiency of surgery.

## Materials and methods

### Patients

This was an analysis of prospectively collected data. All patients who underwent surgery between December 2020 and April 2023 were included. The including criteria were: (1) Papillary thyroid carcinoma was confirmed by pathology; (2) lesion ≤4 cm in the largest dimension without extrathyroidal extension; (3) without clinical evidence of any lymph node (LN) metastases(cN0); (4) The extent of surgery included hemithyroidectomy with prophylactic unilateral central neck dissection(CND). The exclusion criteria were: (1) Intraoperative conversion to open surgery; (2) suspicious malignant lesion in the contralateral lobe; (2) extrathyroidal extend such as recurrent laryngeal nerve, larynx, trachea, esophagus, etc.; (3) hyperthyroidism or sever thyroiditis. The study was approved by the Ethic Committee of Sichuan Cancer Hospital(SCCHEC-03–2018-014).

### Surgical procedure

Laboratory tests included blood routine examination, electrolyte, thyroid function, parathyroid hormone(PTH) and calcitonin was texted before the operation to eliminate surgical contraindications and determine the basal level. Thyroid lesion was assessed by high-resolution ultrasound and contrast-enhanced CT. The assessment of cN0 was determined based on a combination of preoperative examination and intraoperative inspection (12). The procedure included 6 steps (**Supplementary Video 1**): (1) body position and incision design (**Supplementary Figure 1**); (2) building of surgical space (**Supplementary Figure 2**); (3) identification of the inferior parathyroid gland, dissection of the recurrent laryngeal nerve, and central neck dissection (**Supplementary Figure 3**); (4) preservation of the superior parathyroid gland and processing of the superior thyroid vessels (**Supplementary Figure 4**); (5) processing of the suspensory ligament of thyroid gland (**Supplementary Figure 5**); (6) sever the isthmus of thyroid gland and dissect the prelaryngeal lymph nodes (**Supplementary Figure 6**). The updated procedure, operating skills and precautions were described in the **Supplementary File**. The intraoperative neural monitoring(IONM) was utilized in all cases to improve the safety of operation^16^. There is no parathyroid and lymph node development technology during operation.

### Postoperative surveillance

Vital signs and clinical symptoms of patients were monitored in the ward. Patients weren’t given intravenous or oral calcium supplements unless the associated symptoms developed. The electrolytes and parathyroid hormone were determined, and postoperative pain score was evaluated on day 1 by visual analogue scale(VAS). Blood routine examination including white blood cell(WBC), neutrophilic granulocyte(NE), lymphocyte(LYM) and C-reaction protein(CRP) was tested on day 1 and day 2 after surgery. Laryngoscopy would be performed for patients with postoperative persistent abnormal voices.

### Definition

Transient hypoparathyroidism was defined as PTH<15 pg/mL less than six months. The weight of dissection lymphatic tissue(WDLT) was defined as the tissue weight of CND after dissection. The change in postoperative PTH and calcium compared to the preoperative value was calculated as ΔValue=Value(preop)-Value(postop). While the difference between inflammation factors, WBC, NE, LYM and CRP, was calculated as ΔValue=Value(postop)-Value(preop). The values of ΔWBC, ΔNE and ΔCRP indicated the changes of physiological reactions, while the values of ΔLYM indicated the changes of immunological reactions (13).

### Statistical analysis

SPSS 23.0 was used to analyze the collected data, and a *P <*0.05 was considered statistically significant. Continuous variables are described as mean ± SD and/or median(IQR) as appropriate. Categorical variables are described as numbers with percentages. Chi-square or Fisher’s exact test was used to compare categorical variables while t-test or Wilcoxon test was used for continuous variables.

## Results

A total of 302 patients were included in the analysis; of these, 184 patients underwent surgery with the early method, and 118 patients underwent surgery with the updated method. The clinicopathologic characteristics of the patients in the two groups are shown in **Table 1**. There were no significances in terms of sex, age, BMI or rate of Hashimoto thyroiditis. Tumor characteristics were well-balanced between the two groups. The surgical outcomes are shown in **Table 2**. The update method would decrease the operation time(130.4 vs 110 min, respectively; *P*<0.001), time of thyroidectomy with CND(44 vs 41 min, respectively; *P*=0.044), blood loss(20 vs 10 mL, respectively; *P*<0.001), drainage(174 vs 156 mL, respectively; *P*=0.038), postoperative hospital stay(4 vs 3 days, respectively; *P*<0.001).The number of retrieved LNs(3 vs 4, respectively; *P*=0.049) and WDLT(0.75 vs 1.29 g, respectively; *P*<0.001) were more in the update method group than in the early method group with a similar number of positive LNs.

**Table 1:** Clinicopathological characteristics of patients.

	Early(n=184)	Updated(n=118)	*P* Value
Sex, No. (%)			
Male	158(86.9%)	105(89.0%)	0.431
Female	26(14.1%)	13(11.0%)	
Age, year	33 (28,42)	33 (28,41.25)	0.995
BMI	22.5 ± 7.4	21.7 ± 6.7	
Hashimoto thyroiditis, No. (%)	43(23.4%)	35(29.7%)	0.223
Tumor location, No. (%)			
Left	79(42.9%)	50(42.4%)	0.923
Right	105(57.1%)	68(57.6%)	
Tumor size, mm	7(5.9,10)	6.45(5,10)	0.175
T stage, No(%)			
T1	181(98.4%)	118(100%)	0.163
T2	3(1.6%)	0	

**Table 2:** Surgical outcomes and complications.

	Early(n=184)	Updated(n=118)	*P* Value
Operation time, min	130.4(110,150)	110(95,125)	0.000
Space building	23(19,29.8)	22(19,27)	0.082
Thyroidectomy with CND	44(37.3,51)	41(37,48)	0.044
Blood loss, mL	20(10,30)	10(5,20)	0.000
Drainage, mL	174 ± 83.8	156 ± 52.9	0.038
Postoperative hospital stay, day	4(3,5)	3(2,4)	0.000
Complications, No. (%)			
RLN palsy			
Transient	2(1.1%)	5(4.2%)	0.167
Permanent	1(0.5%)	0	1.00
Transient hypoparathyroidism	1(0.5%)	0	1.00
Transient hypocalcemia	24(13%)	17(14.5%)	0.736
Infection	5(2.7%)	1(0.9%)	1.00
Bleeding	5(2.7%)	4(3.4%)	1.00
Superior laryngeal nerve injury	6(3.3%)	3(2.5%)	0.646
Brachial plexus injury	2(1.1%)	0	0.522
Retrieved LNs, No	3(1,5)	4(2,7)	0.049
Metastatic LNs, No	0 (0,1)	0 (0,0.3)	0.074
WDLT, g	0.75(0.45,1.11)	1.29(0.82,1.74)	0.000

CND, central neck dissection; LNs, lymph nodes; RLN, recurrent laryngeal nerve.

WDLT, weight of dissection lymphatic tissue.

There was no significance in postoperative complications between the two groups (**Table 2**). Positional brachial plexus injury occurred in one patient who recovered after functional excise. Permanent RLN palsy occurred in one patient owing to the severing of the RLN, and the immediate repair through end-to-end nerve anastomosis was performed under endoscope. All patients developed transient RLN palsy recovered within 6 months without any specific treatment. No patients who developed postoperative bleeding experienced reoperation.

After dividing each group into 2 subgroups of left hemithyroidectomy with CND and right hemithyroidectomy with CND according to the side of approach, the comparison of surgical outcomes is shown in **Table 3**. The update method still had improvements in operation time(for left side, 128.9 vs 107 min, respectively; *P <*0.001; for right side, 130 vs 113.8 min, respectively; *P*<0.001), blood loss(for left side, 20 vs 10 mL, respectively; P=0.011; for right side, 20 vs 10 mL, respectively; *P*<0.001) and WDLT(for left side, 0.65 vs 1.04 g, respectively; P<0.001; for right side, 0.79 vs 1.48 g, respectively; P<0.001) between different surgery sides. However, no significances were observed at the time of thyroidectomy with CND, drainage and retrieved LNs.

**Table 3:** Surgical outcomes of early and updated methods in different approach sides.

	Early(n=184)	Updated(n=118)	*P* Value
Approach side			
Left	79	50	*-*
Right	105	68	*-*
Operation time, min			
Left side	128.9 ± 28.6	107 ± 23.5	0.000
Right side	130(110,150)	113.8 ± 23.6	0.000
Thyroidectomy with CND			
Left side	44(36,50)	41.6 ± 7.7	0.249
Right side	44(39,51)	43.6 ± 9.4	0.22
Blood loss, mL			
Left side	20(10,20)	10(5,20)	0.011
Right side	20(10,30)	10(5,20)	0.000
Drainage, mL			
Left side	175(140,210)	159 ± 54	0.076
Right side	156(120,190)	152 ± 58	0.458
Retrieved LNs, No			
Left side	2(1,5)	3(1,6)	0.136
Right side	4(2,6.5)	4(3,7)	0.24
WDLT, g			
Left side	0.65(0.4,1.11)	1.04(0.75,1.54)	0.000
Right side	0.79(0.49,1.12)	1.48(0.98,2.04)	0.000

CND, central neck dissection; LNs, lymph nodes; WDLT, weight of dissection lymphatic tissue.

The parathyroid gland preservation and function change are shown in **Table 4**. The results suggested that the update method had more advantages in highlighting superior PG preservation, including reducing the rate of unknown(5.4% vs 0%, respectively; *P*=0.025) and increasing the rate of preservation in situ(92.4% vs 100%, respectively; *P*=0.005), but did not improve the rate of inadvertently removed parathyroid gland and the preservation of inferior parathyroid gland. Meantime, there was no significant between-group difference in the postoperative level change of PTH and calcium as well.

**Table 4:** Parathyroid gland preservation and function change.

	Early(n=184)	Updated(n=118)	*P* Value
Inadvertently removed parathyroid gland	34(18.5%)	21(17.8%)	0.881
Parathyroid preservation, No. (%)			
Superior parathyroid gland			
Preserve in situ	170(92.4%)	118(100%)	0.005
Transplantation	4(2.2%)	0	0.273
Unknown	10(5.4%)	0	0.025
Inferior parathyroid gland			
Preserve in situ	3(1.6%)	4(3.4%)	0.549
Transplantation	90(48.9%)	69(58.5%)	0.104
Unknown	91(49.5%)	45(38.1)	0.054
Preoperative PTH, pg/mL	57.6(43.4,76)	57.4(41.2,77.1)	0.832
ΔPTH	8.3(-6.55,23.67)	5 ± 23.6	0.157
Preoperative calcium mmol/L	2.3(2.25,2.36)	2.28(2.23,2.33)	0.062
Δcalcium	0.09 ± 0.1	0.08 ± 0.11	0.528

PTH, parathyroid hormone.

In terms of surgical trauma (**Table 5**), there was no bias between the 2 methods regarding the baseline of preoperative inflammatory factors values. The values of ΔNE(at day 1, 6.83 vs 6.2 10^9^/L, respectively; *P*=0.01; at day 2, 5.07 vs 4.05 10^9^/L, respectively; *P*<0.001) and ΔCRP(at day 1, 10.27 vs 4.74 mg/L, respectively; *P*=0.001; at day 2, 4.71vs 9.52 mg/L, respectively; *P*=0.003) were significantly lower at both day 1 and day 2 in the update method group than in the early method group, while the values of ΔWBC(5.29 vs 4.36 10^9^/L, respectively; *P*=0.04) and ΔLYM(0.02 vs 0.14 10^9^/L, respectively; *P*=0.011) were significant differences only in day 2. The VAS pain score was less in the update method group(*P*<0.001).

**Table 5:** Surgical trauma.

	Early(n=184)	Updated(n=118)	*P* Value
VAS pain score	2(2,3)	2(2,2)	0.000
Preoperative WBC, 10^9^/L	5.62(4.8,6.48)	5.4 ± 1.22	0.079
ΔWBC day 1	5.96(4.62,8.04)	5.71 ± 2.43	0.45
ΔWBC day 2	5.29(3.77,6.9)	4.36(2.99,6)	0.04
Preoperative NE, 10^9^/L	3.21(2.62,4.06)	3.11 ± 0.89	0.07
ΔNE day 1	6.83 ± 2.68 (-1.75–20.29)	6.2(4.69,7.64)	0.01
ΔNE day 2	5.07(3.72,6.58)	4.05(2.63,5.6)	0.000
Preoperative LYM, 10^9^/L	1.78(1.51,2.12)	1.74(1.48,2.09)	0.344
ΔLYM day 1	-0.71(-0.98,-0.43)	-0.6(-0.88,0.33	0.055
ΔLYM day 2	0.02(-0.28,0.31)	0.14 ± 0.45	0.011
Preoperative CRP, mg/L	0.49(0.49,1.32)	0.65(0.29,1.18)	0.255
ΔCRP day 1	10.27(3.88,12.97)	4.74(2.3,8.59)	0.001
ΔCRP day 2	4.71(2.23,9.74)	9.52 (1.76,5.71)	0.003

WBC, white blood cell; NE, neutrophilic granulocyte; LYM, lymphocyte; CRP, C-reaction protein.

## Discussion

**Figure 1:**
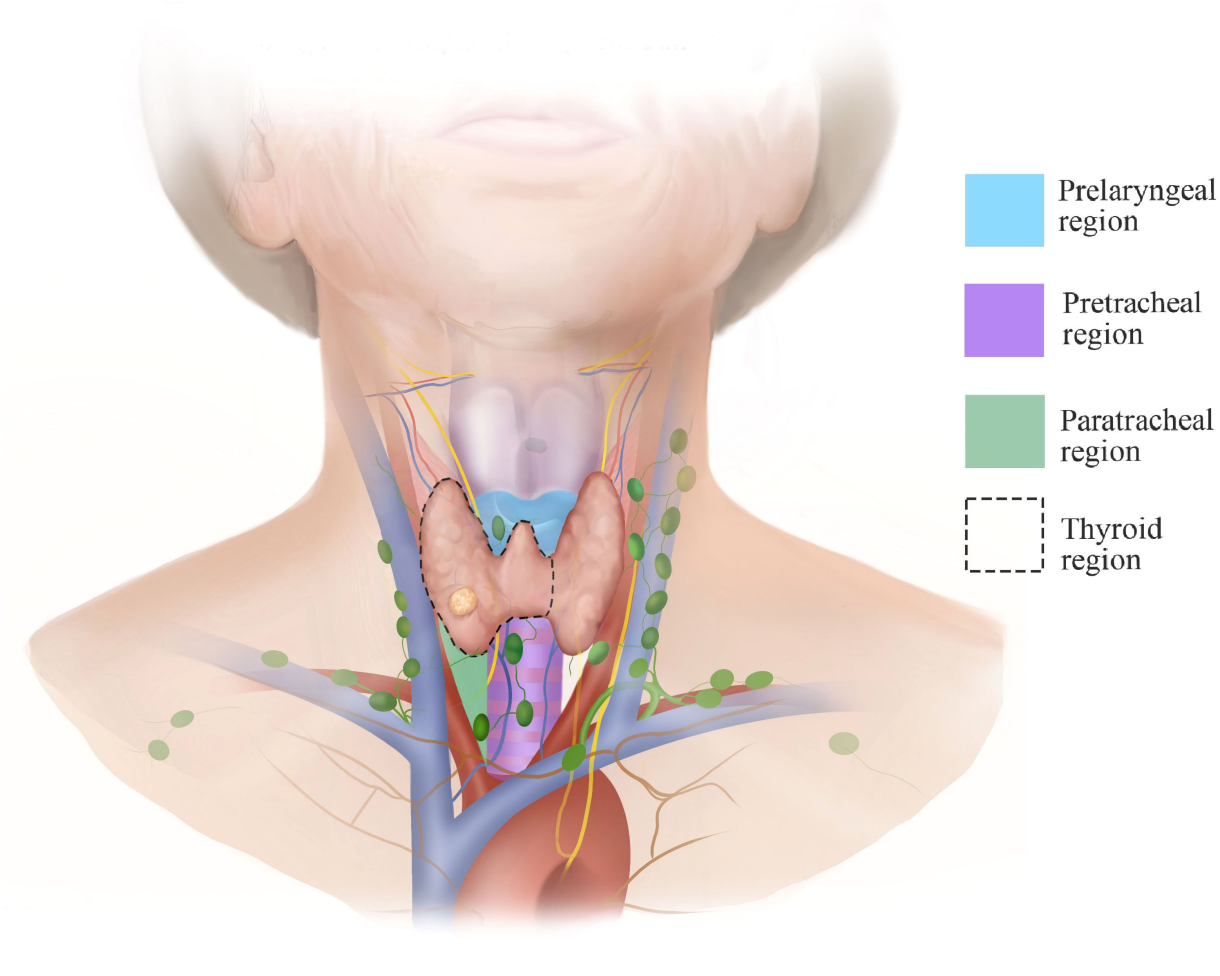
The regions of tumor resection.

In this report, we detail the technical approach to gasless transaxillary endoscopic thyroidectomy for unilateral low-risk thyroid cancer and highlight operating skills and precautions to perform this procedure efficiently without an apparent increased risk of postoperative complications. The steps of tumor resection are regionalized to streamline the procedure. The regions are divided into paratracheal, pretracheal, thyroid gland and prelaryngeal (**Figure 1**). Dissection and protection of structures are performed from one region to another rather than deliberately seeking structures such as the parathyroid glands, the RLN or the trachea and repeatedly separate in multiple areas, which tends to destroy the anatomical integrity at the beginning of the operation.

**Figure 2:**
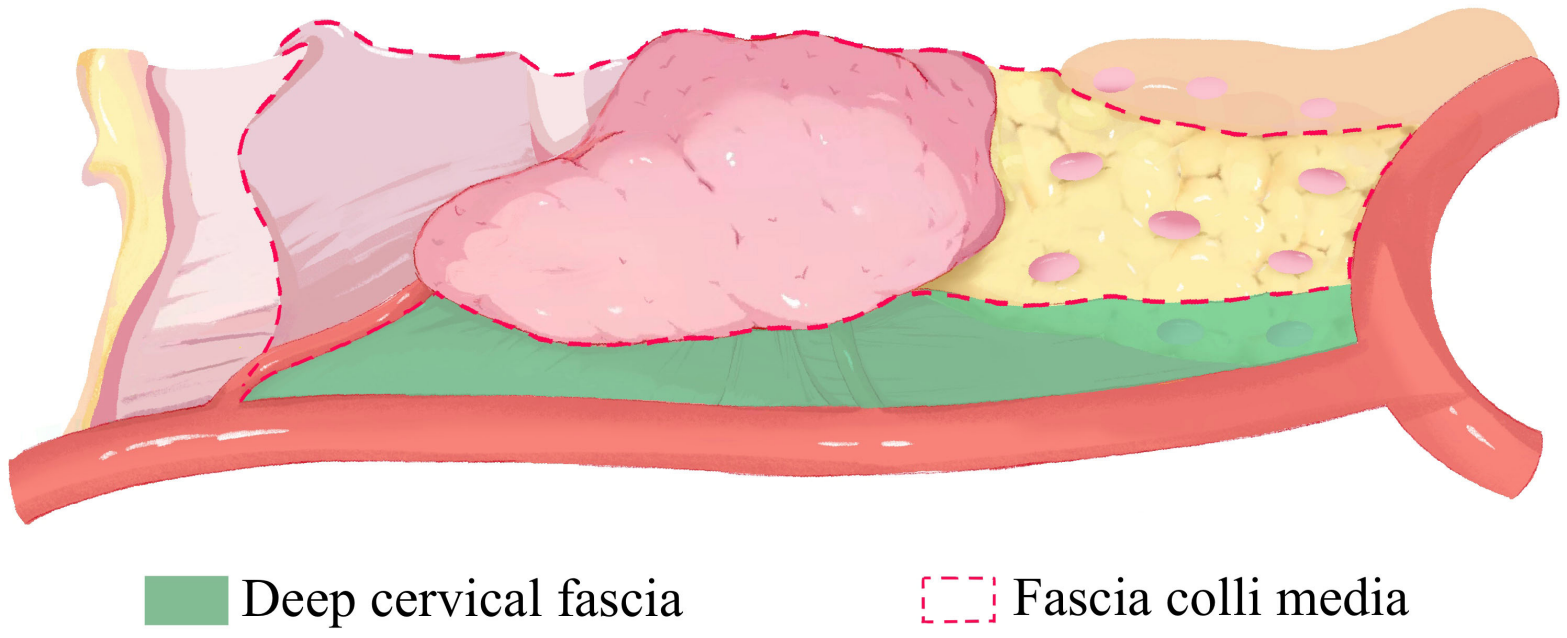
The plane of tumor resection.

**Figure 3:**
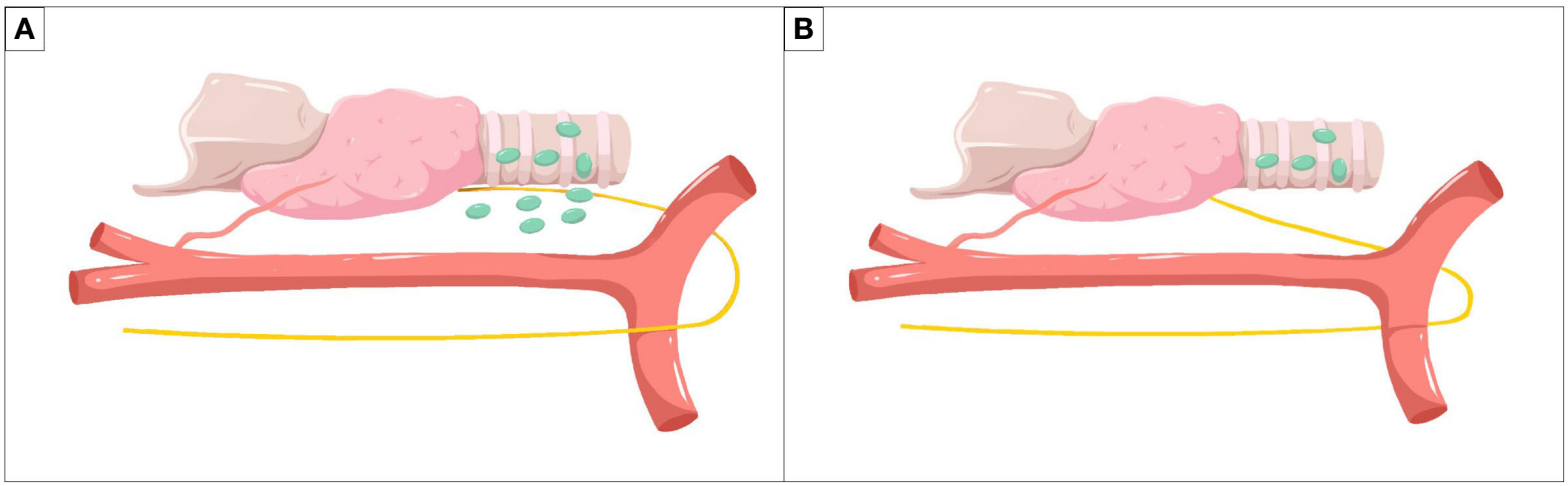
Method of lymph node dissection around the recurrent laryngeal nerve. **(A)** The rise of the recurrent laryngeal nerve for better dissection of deep lymph nodes of the nerve. **(B)** The descent of the recurrent laryngeal nerve for reducing injury to the nerve in subsequent operations.

Meanwhile, the surgical planes (**Figure 2**) are stratified to the deep cervical fascia and the fascia colli media for maintaining the adhesion between levels and exposing the gaps. In this way, the surgical area ascends and remains in the center of view to better maintain the stability of the operation, avoiding excessive movement of the surgical instruments and improving the efficiency of the surgery. Then the operation from deep fascia to media fascia guarantees the lymph nodes are dissected along boundaries and the thyroid membrane, meeting the principle of en block resection and increasing the number of harvested lymph nodes for some patients. With the tension between the RLN and surrounding lymphatic tissue, the nerve is raised and then descended (**Figure 3**). The rise is to better dissection the deep LNs of the nerve, and the descent is to reduce injury to the nerve in subsequent operations.

Our previous experiences and some experts (6–9) gave priority to processing superior and inferior thyroid arteries for reducing bleeding and blurry operating field caused by bleeding. The endoscope technique might contribute to a magnified view of vessels and capillaries facilitating coagulation without significantly increasing bleeding. In some cases, attempts to locate the superior thyroid artery might be hampered by the chromatic aberration of an endoscopic monitor and the adhesion of the thyroid gland to the cricothyroid muscle. It is suggested that the main vessels of thyroid gland were classified as one of the parts in specific regions rather than independent ones.

At the same time, we pay more attention to the flexibility of the operation. The sequence of our surgical steps is not unchanged, which means performing the challenging steps at last. The thyroid ligament is the most frequent site of RLN injury (14)and it is theoretically the last site to deal with in remote-access and open thyroidectomy. In our procedure, whether to sever the ligament in advance depends on the amount of tissue. If the ligament is severed earlier, the cricothyroid space and the superior parathyroid gland will be located more accurately, and the continuity of operation will be enhanced in this method.

We emphasize that the retractors are the core prop of the procedure pulling not only the muscles and skin for maintaining space but also the thyroid gland and lymphatic tissue as grasp forceps. In that way, the mode of dragging the thyroid gland with one hand and repeatedly changing the instruments with another hand like other types of remote-access thyroid surgery (15–17) will be replaced by a two-hand manipulation, shortening the time of thyroidectomy and lymph node dissection. The retractors will be invisible assistants through exposing the gaps and adjusting the height of structures dynamically, and the difficulties of some steps will be reduced. In addition, the cover of paratracheal area by the SCM or carotid sheath will be less through flexible use of retractions, and at the same time, the pain even trauma caused by excessive pulling to neck with a retractor for more exposure will be reduced.

Our method has limitations. First, the ideal indication of our method is adult patients with papillary thyroid carcinoma(TNM stage T1-T2, N0) as the recommendation of the American Thyroid Association statement (18). Whether our method is suitable for patients with preoperative lymph node metastasis to level VI(cN1a) or T3b node-negative PTC needs to be further explored. Second, owing to uncertain components retained in the position of PG, our steps haven’t addressed the question of preservation of the inferior PG *in situ*, whether it will improve through the auxiliary intraoperative parathyroid identification or protection techniques remains uncertain.

## Conclusion

Gasless transaxillary endoscopic thyroidectomy has been popular among surgeons for its unique advantages. It is hoped that our improvements in surgical procedures will contribute to the progress and popularization of this approach.

## Data availability statement

The raw data supporting the conclusions of this article will be made available by the authors, without undue reservation.

## Author contributions

YZ: Data curation, Writing – original draft, Writing – review & editing, Conceptualization, Formal Analysis, Project administration. CS: Conceptualization, Data curation, Formal Analysis, Writing – original draft. LM: Project administration, Writing – review & editing, Data curation. YC: Conceptualization, Writing – review & editing. RS: Project administration, Writing – review & editing. JJ: Conceptualization, Writing – review & editing. DZ: Formal Analysis, Writing – review & editing. XW: Data curation, Writing – review & editing. XX: Data curation, Writing – review & editing. PH: Data curation, Writing – review & editing. CL: Conceptualization, Investigation, Methodology, Project administration, Supervision, Writing – review & editing.
