# Supplementary material for: The updated surgical steps of gasless transaxillary endoscopic thyroidectomy with neck level and region orientation for thyroid cancer
Source: Front Oncol. 2024 May 10;14:1377878. doi: 10.3389/fonc.2024.1377878 (PMC11116616; doi:10.3389/fonc.2024.1377878)
Supplement: Supplementary file 1 [file DataSheet_1.docx]

**Step 1: body position and incision design**

Under general anesthesia, the patient is placed in the supine position on a pad positioner, with chin up and head tilted about 45º to the healthy side. The affected upper limb is naturally abducted 90–180°(supplemental figure 1). If the clavicle at the affected side is higher than the thyroid isthmus under direct visualization, the angle of the upper limb abduction is appropriately adjusted to higher than 90° until the clavicle is inferiorly displaced below the level of the thyroid isthmus. After the axilla at the affected side is exposed, an incision is made under the natural folds in the axilla. Cut open the subcutaneous fatty layer until the upper lateral edge of the pectoralis major muscle is exposed.

*Operating skills and precautions:*

(1) The chin-up will stretch the neck to expose the central neck compartment better.

Excessive upper limb abduction should be avoided positional brachial plexus^[1]^ or joint injury resulting in postoperative shoulder and upper limb discomfort.

(2) There are about two to four folds in the axilla. It will be better for incision along the fold, as it affects the aesthetic outcome. The incision close to the head side has a good view but the chopstick effect is obvious. On the contrary, the incision close to the foot side is convenient for operation but the view is limited. The incision should not exceed the anterior axillary line because it cannot be covered when the arm falls naturally.

(3) An operation along the anterior axillary line is more convenient but is associated with poorer aesthetic outcomes.

**Step 2:** **building of surgical space**

The building of surgical spaces mainly follows the three-step method involving lifting the surface skin of the pectoralis major muscle to create the chest wall cavity, lifting the sternocleidomastoid muscle or its sternal head to create the carotid sheath cavity, and lifting the strap muscle to create the thyroid cavity. The space is separated from the 2 ends of the incision to suprasternal fossa (lower border) and pyramidal lobe of thyroid(upper border). The specific steps are as follows: A long-headed electric knife separates the gap pulled by an assistant between the muscle and subcutaneous fat to the clavicle. The integrity of the membrane of the pectoralis major muscle is maintained during the division to reduce muscle injury and bleeding and keep the view clear. When the electric knife is unable to continue the division, the insertion of a suspension retractor is used to maintain the space. A trocar is placed in the axillary incision about two fingers away from the breast side and slightly below the anterior axillary line, and then start the endoscopic operation. The subcutaneous tunnel is separated along the demarcated area by using an electrocautery or ultrasonic scalpel(supplemental figure 2A). Do not overemphasize the integrity of the membrane so separate too shallowly to skin perforation. The supraclavicular nerve, a branch of the cervical plexus nerve, should be carefully protected when the separation crosses the clavicle to reduce postoperative numbness in the supraclavicular region. The sternocleidomastoid muscle(SCM) bundle will be exposed when the separation crosses the clavicle, and the posterior border of the SCM can be visible with a little separation. The yellowish-white natural gap between the sternal and clavicular heads of the SCM(supplemental figure 2B) or the posterior edge of the muscle is exposed naturally or after retraction with an endoscopic device. The fascial tissues are separated along the natural gap, and the sternal head or the posterior edge of the muscle is lifted by moving the suspension hook. After the second space is built, the omohyoid muscle and the carotid sheath are exposed. The omohyoid muscle is an important anatomical landmark(supplemental figure 2C), with the carotid sheath and the strap muscle on its deep surface. A “muscular triangle” is formed at the junction between the lateral side of the strap muscle and the omohyoid muscle, and the thyroid gland is exposed by separation along the gap between the lateral side of the strap muscle(supplemental figure 2D) and the carotid sheath. Whether the omohyoid muscle is preserved or not depends on the operator’s proficiency and preference or depends on the exposure of the surgical field. The lateral border of the strap muscle is identified through the muscular triangle. The deep side of the strap muscle is separated for straight exposure of the deep side of the glandular fascia(supplemental figure 2E), and the retractor is advanced to lift the strap muscle to complete the third step of space building. The 2 sides of the sternocleidomastoid muscle are appropriately loosened and lowered according to the visibility of the intraoperative field.

*Operating skills and precautions:*

(1) The insertion site of trocar can also be adjusted according to the actual situation or personal preferences. The farther the site is away from the axillary incision, the less obvious the “chopstick effect” (i.e., the interference among multiple instruments) is.

(2) Nevertheless, the puncture point should be kept above the pectoralis major muscle to avoid the obstruction of the instrumentation by the muscle.

(3) When the separation passes across the clavicle, there is no pectoralis major muscle in the field of view, but adipose and fascial connective tissues. Excessive separation towards the deep side may cause brachial plexus injuries and ipsilateral upper limb lymphedema. The separation gap can be exposed better by increasing the pulling force of the retractor during separation.

(4) The identification of a gap in the sternocleidomastoid muscle is one of the difficulties in space building for obscure in some patients.

(5) It is necessary to carefully distinguish the distribution of muscle bundles or to search for by using separation forceps.

(6) Unfortunately, the muscles abundant with bundles will mislead dissection into an incorrect plane. It is time to identify the gap by searching for the plexus vacuous of the muscle and adipose tissue in the muscle area. The gap can also be stretched out by pulling on the uppermost edge of the sternocleidomastoid muscle in the visual field with a retractor.

(7) For some patients whose the gap is not anatomically obvious or in whom exposing the gap is difficult, the posterior edge of the sternocleidomastoid with a relatively fixed anatomy can be an ideal approach alternative^[2]^. The external jugular vein should be carefully protected when the posterior edge of the sternocleidomastoid approach is adopted.

(8) During separation of the gap in the sternocleidomastoid muscle, the distance between the 2 sides of the separation can be slightly larger than the width of the retractors. When the separation proceeds to the cephalad side, any injury to the external jugular vein should be avoided, with active avoidance being the best option. (IV) The strap muscle is composed of the sternohyoid muscle and the sternothyroid muscle. Both muscles can be lifted together for separation, which avoids further bleeding and trauma caused by separating them one by one. For some patients with severe hashimoto thyroiditis, the effect of direct separation is not obvious and will cause the oozing of blood for tight adhesion of thyroid gland and the strap muscle. The gap can be separated into multiple points like a “net”, and then these points are connected into a plane by an ultrasonic scalpel.

(9) In addition, excessive separation of the strap muscle to thyroid isthmus should be avoided. The separation is sufficient if the retractor can be inserted, which allows for the traction of the gland by the connection between the gland and the muscle and facilitates traction of the recurrent laryngeal nerve(RLN) to the surface of the trachea, exposure of deep side of the RLN of the tracheoesophageal groove and central neck dissection(CND).

**Step 3: identification of the inferior parathyroid gland, dissection of the recurrent laryngeal nerve, and central neck dissection**

As the en bloc resection of the tumor is required during the surgery, central neck dissection is performed before thyroidectomy; but the inferior parathyroid glands must be identified before lymph node dissection. Typically, the location of the inferior parathyroid gland(PG) is highly variable with most glands (approximately 80%) located in the area between the lower pole of the thyroid gland and the thymus^[3]^ and is more difficult to preserve in situ. So that the inferior PG should be searched for in the middle and upper portions of the visual field. Auto transplantation should be adopted decisively for the inferior PG that cannot be preserved in situ and hasn’t stable blood supply or misresction. Blunt separation in the middle and lower of paratracheal area is performed to expose the RLN(supplemental figure 3A) and (supplemental figure 3B) (the latter is vertical across the deep side of the RLN). Dissect the medial edge of the common carotid artery and expose the lateral border of the central neck compartment^[4]^. The inferior artery of thyroid and its surrounding veins are coagulated below the RLN. Owing to the gland and tissue in the central neck pulled upward by a suspension hook, the deep lymph nodes of the RLN(level VIB) are fully exposed. Losing the adhesion between the RLN and the lymphatic tissues in level VIB, the RLN will be pulled to the top of the surgical field. Then the dissection of level VIB can be performed along the lateral border of the central neck compartment(supplemental figure 3C). After losing the adhesion of the RLN and its superficial lymphatic tissue(level VIA), lymphatic tissue in level VIA will be lifted by the strap muscles while the RLN will descend into the area of the tracheoesophageal groove(supplemental figure 3D). Meanwhile, the trachea was located on the medial side of the view. Paratracheal lymphatic tissue is separated into the pretracheal area along the surface of trachea(supplemental figure 3E). After the lymphatic tissue is pulled to head side to full exposure, the lymph nodes of pretrachea are dissected along the inferior and medial borders.

*Operating skills and precautions*

(1) The coagulated end of the inferior thyroid artery should be located in the surgical field of view to facilitate the procession of bleeding.

(2) The retractors pulled off the muscles and the thyroid gland for better exposure of the central neck compartment but caused the start position of thyroidectomy lower than that of the inferior PG. In other words, there were few chances of preserving inferior PG in situ when the RLN was dissected or lymph node dissection started. Auto transplantation seems to be acceptable given that there was no risk factor associated with transient hypoparathyroidism in multivariate analysis based on thyroid lobectomy^[5]^

(3) The lymph nodes at the thoracic inlet are challenging, resulting in difficult exposure or a severe chopstick effect. Thus, the positions of the instruments and laparoscope need to be adjusted constantly and the pull of lymphatic tissues should be maintained. Preoperative computed tomography (CT) and other imaging assessments in this area are particularly important.

(4) The thymus should be protected during the dissection of the pretracheal lymph nodes to prevent the misresection of the heterotopic parathyroid gland or damage of the blood supply of the inferior parathyroid gland, nevertheless, attention should also be paid to the occult lymph nodes beneath the thymus^[6]^.

(5) Exposure is regarded as the gold standard way to protect RLN during thyroid surgery^[7,8]^. The exposed RLN should always be located in the visual field when operating around the nerve. The intraoperative nerve monitoring system can also be used to assist in the identification and protection of the nerve^[7]^.

(6) The working head of the energy instruments should be kept away from the RLN when dissecting the perineural tissues, with a safe distance of at least 3–5 mm^[9]^. Even if the energy instruments have a safe work distance, it is still recommended to operate around the RLN with sharp or cold devices.

(7) Oozing of blood from the capillaries around the RLN will be magnified by the endoscope. Pressure with a small strip of wet gauze can greatly reduce oozing instead of energy devices.0

(8) In some cases, the thyroid veins are distributed in the deep side of the pretracheal tissue. It is safe to isolate and coagulate these vessels respectively or to dissect the area at low-power mode of energy instruments.

**Step 4: preservation of the superior parathyroid gland and processing of the superior thyroid vessels**

Bluntly dissect the RLN along the route of anatomy towards the nerve entry into the larynx. Don’t rush the suspensory ligaments of thyroid gland unless it’s very thin. Pass over it and continue to separate close to the thyroid capsule towards the head side for searching the upper parathyroid gland. The superior parathyroid glands have relatively fixed locations^[3]^. The superior region of the gland is finely dissected by separating the true and false peritonea, for protecting the superior parathyroid glands and their surrounding vascular networks(supplemental figure 4A). Seek the cricothyroid space in the plane of deep side of the gland(supplemental figure 4B), and separate towards the region of superior thyroid vessels. After the superior polar thyroid gland is separated and completely exposed along the cricothyroid space, the superior thyroid vessels can be skeletonized from different directions(supplemental figure 4C). Pulling the gland towards the foot side can expose the superior blood vessels better. The ultrasonic scalpel at multiple points reduces the risk of bleeding and avoids damage to the cricothyroid muscle or superior laryngeal nerve. The superior laryngeal nerve can also be identified beneath the superior thyroid vessels, dissected, and protected first before coagulation of the superior vessels. The intraoperative nerve monitoring system can be used to assist in identifying and protecting the superior laryngeal nerve.

*Operating skills and precautions:*

(1) The separation close to the thyroid capsule should be ensured to avoid mistaking the cricothyroid muscle for thyroid gland and causing muscle injury.

(2) There are many tiny vessels and dense tissues at the RLN laryngeal entry point, and bleeding caused by hard separation with endoscopic instruments should be avoided.

(3) A wet gauze strip can be used to push the RLN downwards to expose the gap around the nerve laryngeal entry point

(4) For patients with a high position of the superior thyroid vessels and large thyroid gland, the exposure of the superior pole vessels may be difficult; in such cases, a small amount of the strap muscle can be further separated towards the head (with the muscle being cut off if necessary).

(2) During the processing of the superior pole thyroid gland, the omohyoid muscle can also be cut off if it covers the surgical field.

**Step 5:** **processing of the suspensory ligament of thyroid gland**

Whether to perform the step 4 or the step 5 depends on the amount of tissue in the thyroid ligament. The dense tissue in the suspensory ligament is reduced after dissociating the surrounding glands(supplemental figure 5A). Bipolar electrocoagulation followed by cutting can be considered to minimize thermal injury and bleeding. The suspensory ligament should be dissected in a single attempt or layer by layer(supplemental figure 5B), as multiple attempts may result in a residual gland. A wet gauze strip is used to shield the RLN laryngeal entry point to reduce thermal injury.

*Operating skills and precautions:*

(1) In some patients, a small number of glands may be preserved at the suspensory ligament as appropriate to ensure postoperative voice function and quality of life.

**Step 6: sever the isthmus of thyroid gland and dissect the prelaryngeal lymph nodes**

After the above steps, the gland is mostly mobilized and pulled up, allowing better exposure of the pretracheal space. The gland which extends from the inferior pole to superior pole of thyroid is separated in the pretracheal space to the thyroid isthmus(supplemental figure 6A). The isthmus of thyroid gland is severed along the pretracheal space(supplemental figure 6B). Then the patient’s head is tilted to the operator’s side, and the prelaryngeal lymph nodes and pyramid lobe are dissected(supplemental figure 6C). Finally, The excised tumor and lymph nodes are removed as a whole from the axillary incision. The surgical cavity is irrigated. Adequate hemostasis is required, as the sternocleidomastoid muscle, clavicle, and supraclavicular fossa are easy to ooze blood. A drainage tube is placed through the axillary incision. The axillary incision is sutured layer by layer. Finally, a bandage with pressure is applied in the axillary, supraclavicular, and cervical areas.

*Operating skills and precautions:*

(1) By tilting the patient’s head to the side of the operator, the cover of thyroid pyramidal lobe and the prelaryngeal lymph nodes by the larynx will be reduced.

(2) Despite the large size of axillary space, direct removal of the specimens from the axillary cavity should be avoided, and transport by using a specimen bag can reduce the risk of thyroid or tumor implantation.

**References**

1. Stang MT, Yip L, Wharry L, Bartlett DL, McCoy KL, Carty SE. Gasless Transaxillary Endoscopic Thyroidectomy with Robotic Assistance: A High-Volume Experience in North America. Thyroid(2018) 28(12):1655-1661. doi: 10.1089/thy.2018.0404.
2. Zhou YQ, Li C, Cai YC, Jiang J, Sun RH, Zeng DF, et al. Posterior sternocleidomastoid border approach of gasless transaxillary endoscopic thyroidectomy in patients with papillary thyroid carcinoma: comparison with sternocleidomastoid fascia approach. Zhonghua Wai Ke Za Zhi(2021) 59(8):686-690. doi: 10.3760/cma.j.cn112139-20200817-00651.
3. Zhu J, Tian W, Xu Z, Jiang K, Sun H, Wang P, et al. Expert consensus statement on parathyroid protection in thyroidectomy. Ann Transl Med(2015) 3(16):230.
4. Agrawal N, Evasovich MR, Kandil E, Noureldine SI, Felger EA, Tufano RP, et al. Indications and extent of central neck dissection for papillary thyroid cancer: An American Head and Neck Society Consensus Statement. Head Neck(2017) 39(7):1269-1279. doi: 10.1002/hed.24715.
5. Xing Z, Qiu Y, Fei Y, Xia B, Abuduwaili M, Zhu J, et al. Protective strategy of parathyroid glands during thyroid lobectomy: A retrospective cohort and case-control study. Medicine (Baltimore)(2021) 100(14):e21323.
6. Li W, Wang B, Jiang ZG, Feng YJ, Zhang W, Qiu M. The role of thymus preservation in parathyroid gland function and surgical completeness after bilateral central lymph node dissection for papillary thyroid cancer: A randomized controlled study. Int J Surg(2019) 65:1-6. doi: 10.1016/j.ijsu.2019.02.013.
7. Randolph GW, Dralle H; International Intraoperative Monitoring Study Group; Abdullah H, Barczynski M, Bellantone R, Brauckhoff M, Carnaille B, et al. Electrophysiologic recurrent laryngeal nerve monitoring during thyroid and parathyroid surgery: international standards guideline statement. Laryngoscope. 2011;121 Suppl 1:S1-16. doi: 10.1002/lary.21119.
8. Biswas SS, Hossain MM, Mahbub S, Razib SF. Routine Exposure Versus Non-Exposure of Recurrent Laryngeal Nerve during Thyroid Surgery: Our Experience of 300 Cases. Mymensingh Med J(2022) 31(1):154-160.
9. Jiang H, Shen H, Jiang D, Zheng X, Zhang W, Lu L, et al. Evaluating the safety of the Harmonic Scalpel around the recurrent laryngeal nerve. ANZ J Surg(2010) 80(11):822-826. doi: 10.1111/j.1445-2197.2010.05436.x.

Supplementary Figure 1. Body position and incision design

Supplementary Figure 2. Building of surgical space.

A. Separation of the pectoralis major muscle ﬂap.

B. Identification of the sternocleidomastoid muscle gap.

C. Dissection of the omohyoid muscle.

D. Separation lateral side of the strap muscle.

E. Traction of the strap muscle to expose the thyroid.

Supplementary Figure 3. Dissection of the recurrent laryngeal nerve, and central neck dissection.

A. Exposure of the recurrent laryngeal nerve.

B. Exposure of the inferior thyroid artery.

C. Dissection of lymph nodes of the deep side of the recurrent laryngeal nerve.

D. Descent of the recurrent laryngeal nerve.

E. Dissection of lymph nodes of the superficial side e of the recurrent laryngeal nerve.

Supplementary Figure 4. Preservation of the superior parathyroid gland and processing of the superior thyroid vessels.

A. Protection of the superior parathyroid glands and surrounding vascular networks.

B. Seek the cricothyroid space.

C. Exposure to superior thyroid vessels.

Supplementary Figure 5. Processing of the suspensory ligament of thyroid gland.

A. Reduction of dense tissue around the thyroid gland.

B. Dissection of the suspensory ligament.

Supplementary Figure 6. Sever the isthmus of thyroid gland and dissect the prelaryngeal lymph nodes.

A. Separation of gland to the thyroid isthmus

B. Severing the thyroid isthmus.

C. Dissection of the prelaryngeal lymph nodes and the pyramid lobe.
