# Supplementary figures and images for: The updated surgical steps of gasless transaxillary endoscopic thyroidectomy with neck level and region orientation for thyroid cancer
Source: Front Oncol. 2024 May 10;14:1377878. doi: 10.3389/fonc.2024.1377878 (PMC11116616; doi:10.3389/fonc.2024.1377878)

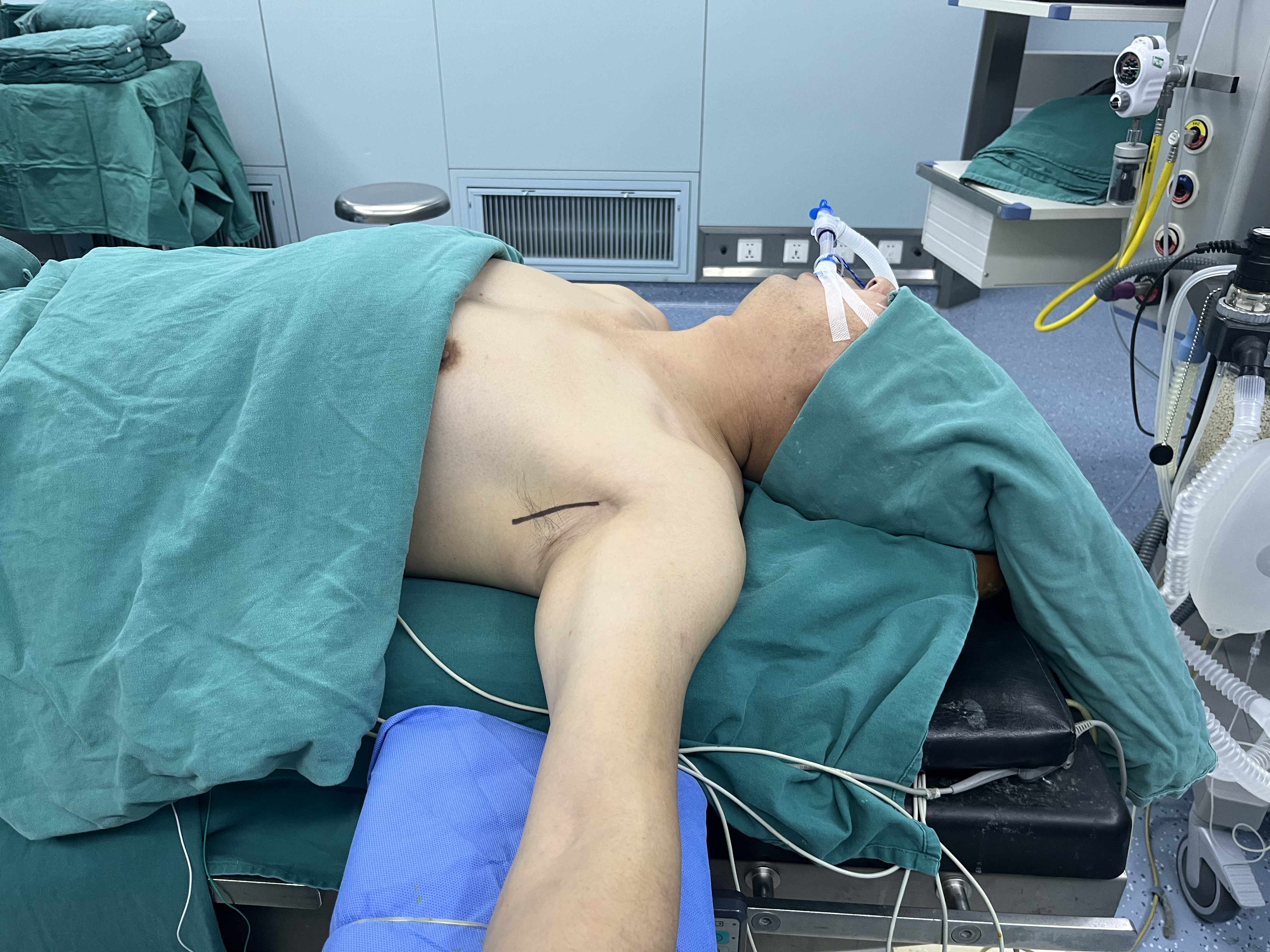

Supplement: Supplementary file 2 [file Image_1.jpeg]

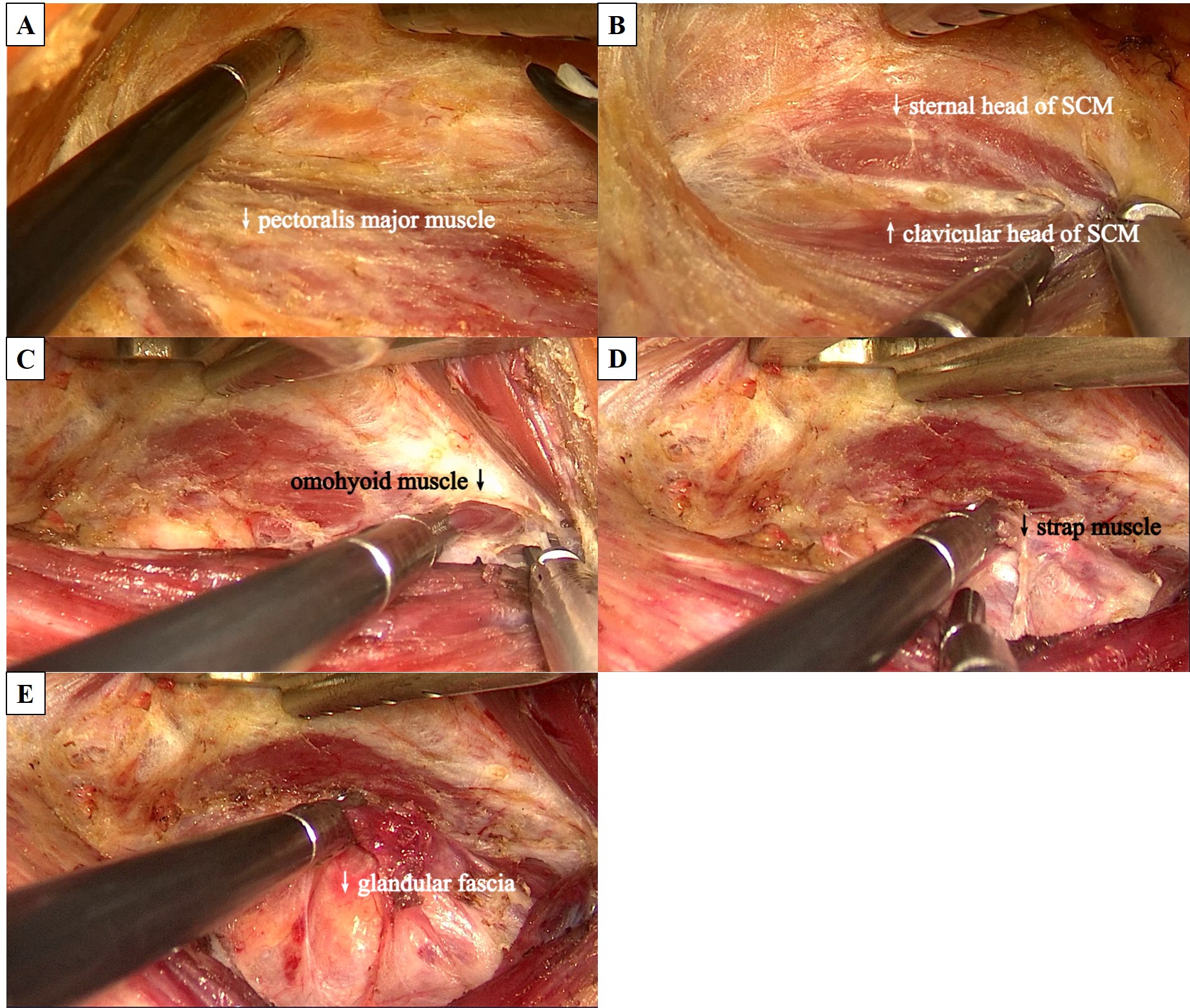

Supplement: Supplementary file 3 [file Image_2.jpeg]

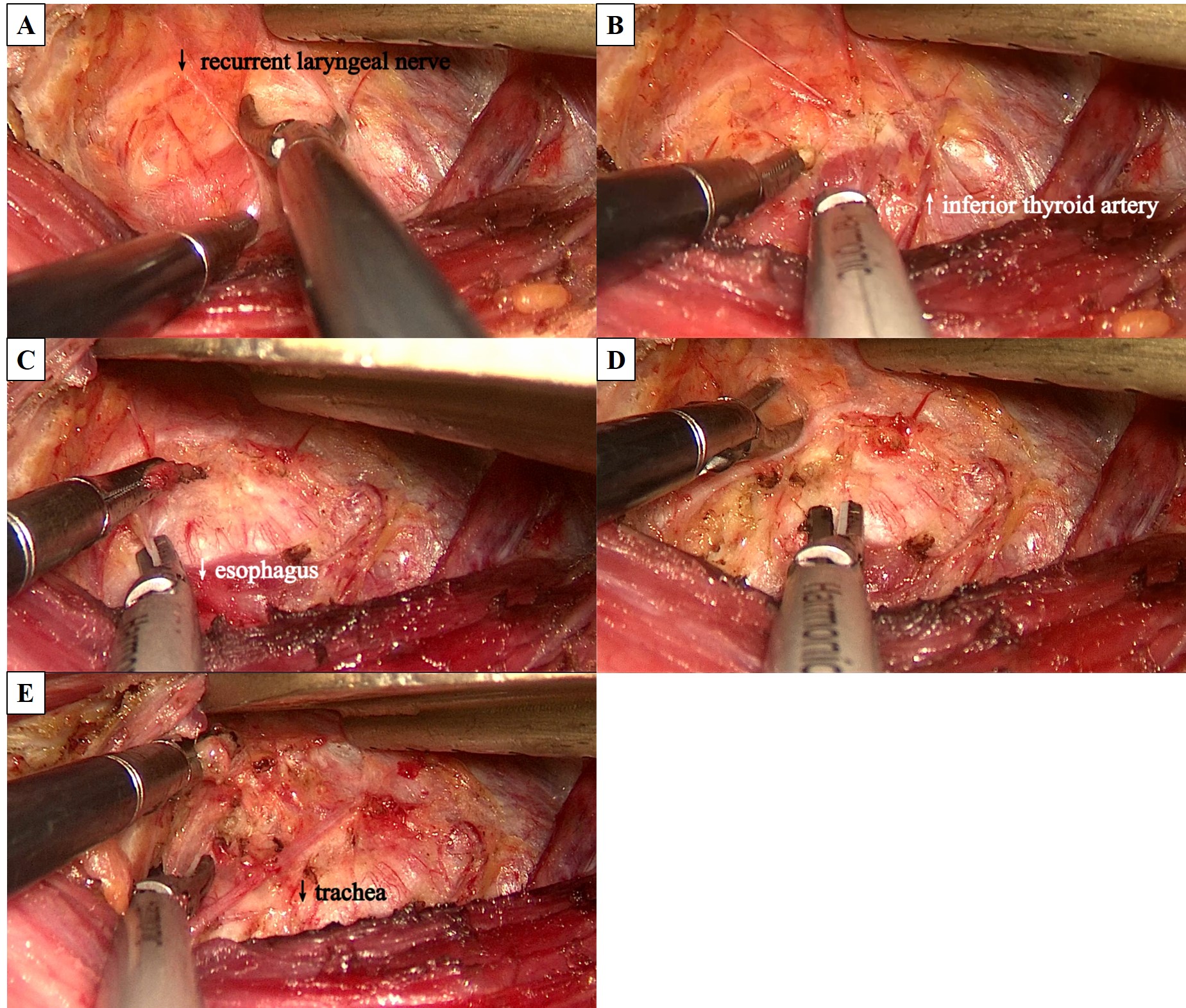

Supplement: Supplementary file 4 [file Image_3.jpeg]

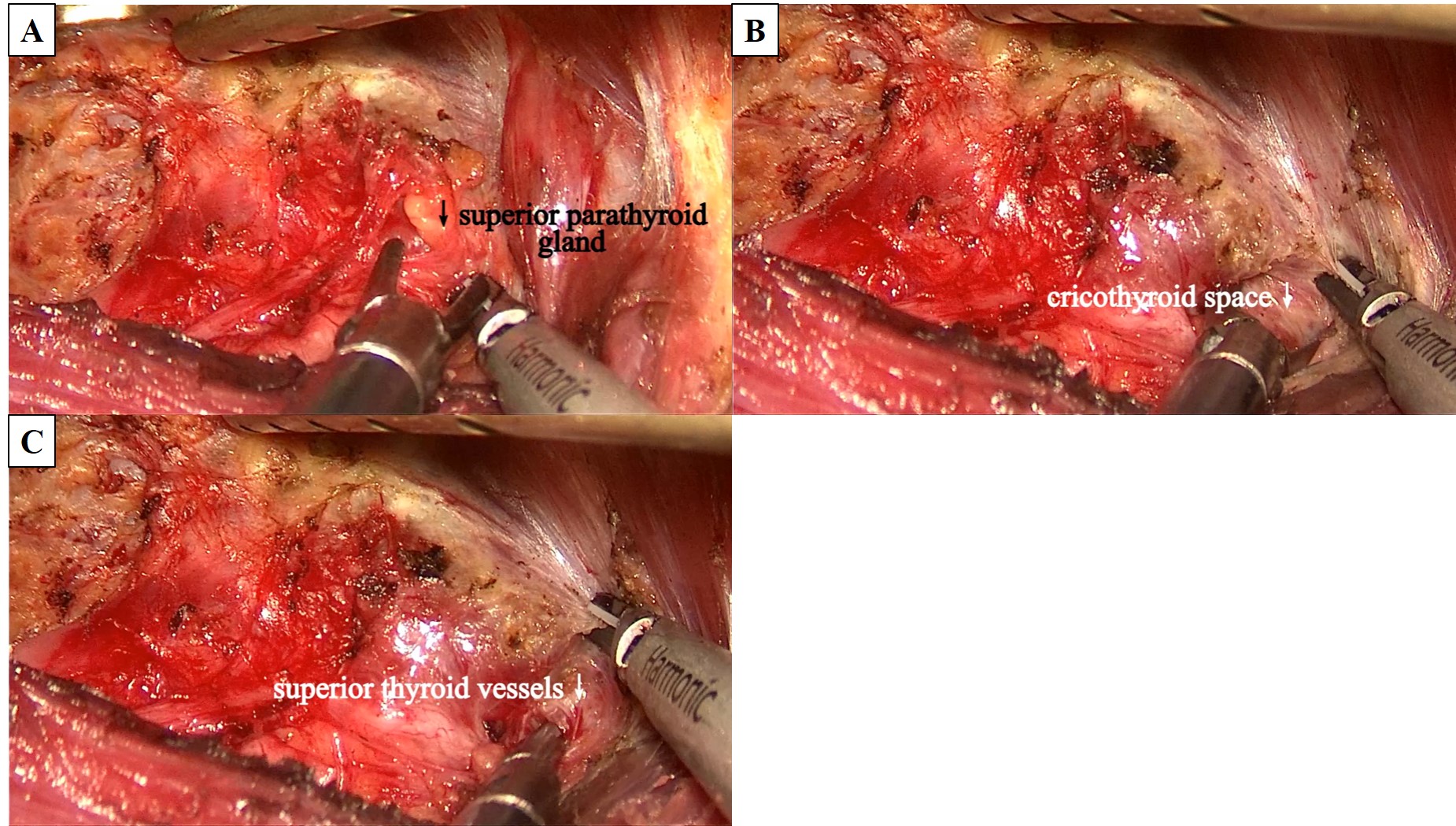

Supplement: Supplementary file 5 [file Image_4.jpeg]

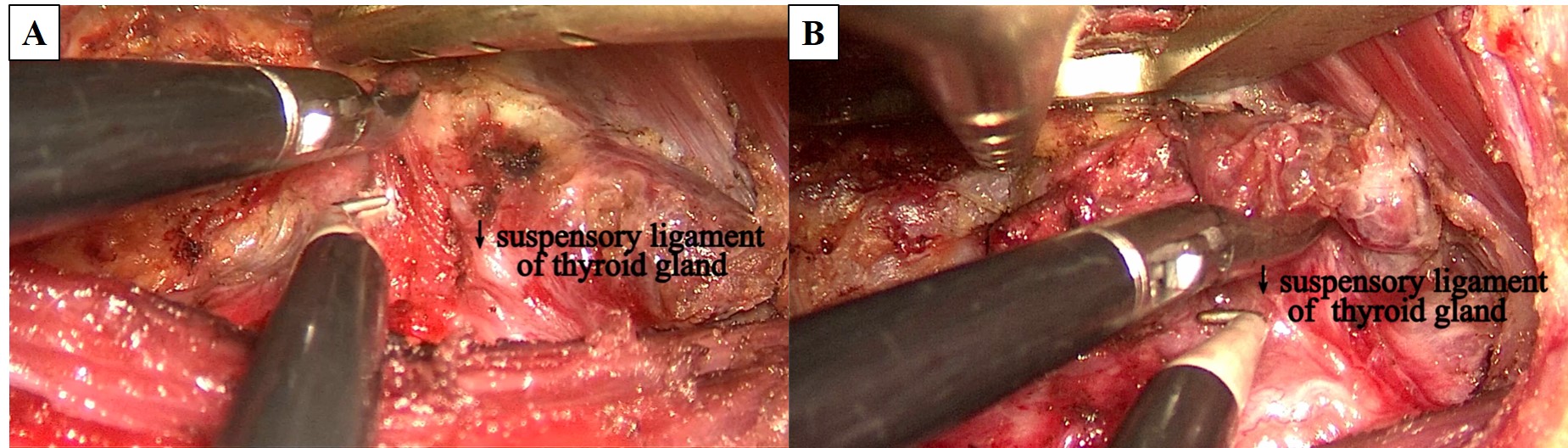

Supplement: Supplementary file 6 [file Image_5.jpeg]

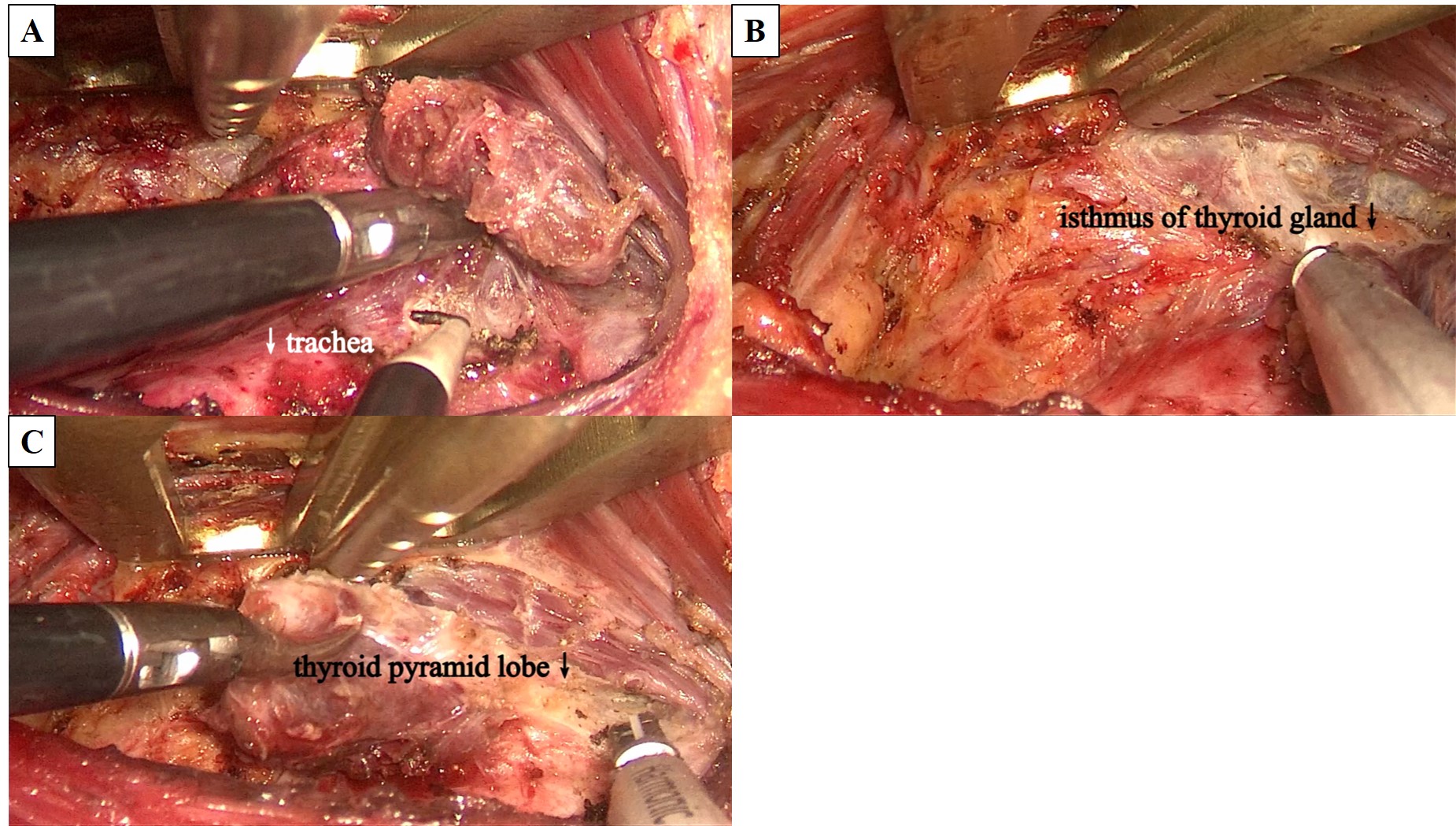

Supplement: Supplementary file 7 [file Image_6.jpeg]
